# Matrix metalloproteinase-12 is an essential mediator of acute and chronic arterial stiffening

**DOI:** 10.1038/srep17189

**Published:** 2015-11-26

**Authors:** Shu-Lin Liu, Yong Ho Bae, Christopher Yu, James Monslow, Elizabeth A. Hawthorne, Paola Castagnino, Emanuela Branchetti, Giovanni Ferrari, Scott M. Damrauer, Ellen Puré, Richard K. Assoian

**Affiliations:** 1 Department of Systems Pharmacology and Translational Therapeutics, University of Pennsylvania, Philadelphia, PA 19104; 2 Department of Biomedical Sciences, University of Pennsylvania, Philadelphia, PA 19104; 3 Department of Surgery, University of Pennsylvania, Philadelphia, PA 19104

## Abstract

Arterial stiffening is a hallmark of aging and risk factor for cardiovascular disease, yet its regulation is poorly understood. Here we use mouse modeling to show that matrix metalloproteinase-12 (MMP12), a potent elastase, is essential for acute and chronic arterial stiffening. MMP12 was induced in arterial smooth muscle cells (SMCs) after acute vascular injury. As determined by genome-wide analysis, the magnitude of its gene induction exceeded that of all other MMPs as well as those of the fibrillar collagens and lysyl oxidases, other common regulators of tissue stiffness. A preferential induction of SMC MMP12, without comparable effect on collagen abundance or structure, was also seen during chronic arterial stiffening with age. In both settings, deletion of MMP12 reduced elastin degradation and blocked arterial stiffening as assessed by atomic force microscopy and immunostaining for stiffness-regulated molecular markers. Isolated MMP12-null SMCs sense extracellular stiffness normally, indicating that MMP12 causes arterial stiffening by remodeling the SMC microenvironment rather than affecting the mechanoresponsiveness of the cells themselves. In human aortic samples, MMP12 levels strongly correlate with markers of SMC stiffness. We conclude that MMP12 causes arterial stiffening in mice and suggest that it functions similarly in humans.

The biomechanical properties of arteries and their extracellular matrix (ECM) play a critical role in cardiovascular disease (CVD). Arteries stiffen with age, and arterial stiffening is a cholesterol-independent risk factor for CVD^1^,^2^,^3^,^4^,^5^,^6^. Arterial stiffening increases endothelial permeability^7^, macrophage adhesion^8^, smooth muscle proliferation^9^,^10^, and vessel remodeling^11^. Thus, therapies that could limit arterial stiffening would be highly valued and likely complementary to existing pharmacological interventions that treat CVD by lowering blood cholesterol. However, development of these new therapies requires currently lacking knowledge about the dynamic regulators of arterial stiffness.

Arterial stiffness is determined by changes in vascular tone and the composition of the arterial extracellular matrix (ECM). Vascular tone is largely controlled by endothelial-derived nitric oxide and PGI_2_, which regulate contractility of differentiated SMCs in a paracrine manner^12^. However, differentiated SMCs modulate to a dedifferentiated state in CVD^13^,^14^. These dedifferentiated cells show reduced expression of differentiation-specific contractile proteins such as smooth muscle-α actin (SMA) and smooth muscle myosin heavy chain but increase their production of ECM components that remodel the arterial ECM. Remodeling of the arterial ECM can lead to arterial stiffening, which is thought to reflect changes in the synthesis of ECM proteins as well as degradation of the ECM by matrix metalloproteinases (MMPs)^11^,^15^,^16^. Here, we show that acute and chronic arterial stiffening in mice is accompanied by a striking induction of MMP12, a potent elastase, in vascular SMCs. We also show that MMP12 is essential for arterial stiffening in mice and is a highly prognostic marker of arterial stiffness in humans.

## Results

### Smooth muscle MMP12 and acute arterial stiffening in the response to vascular injury

**Figure 1:**
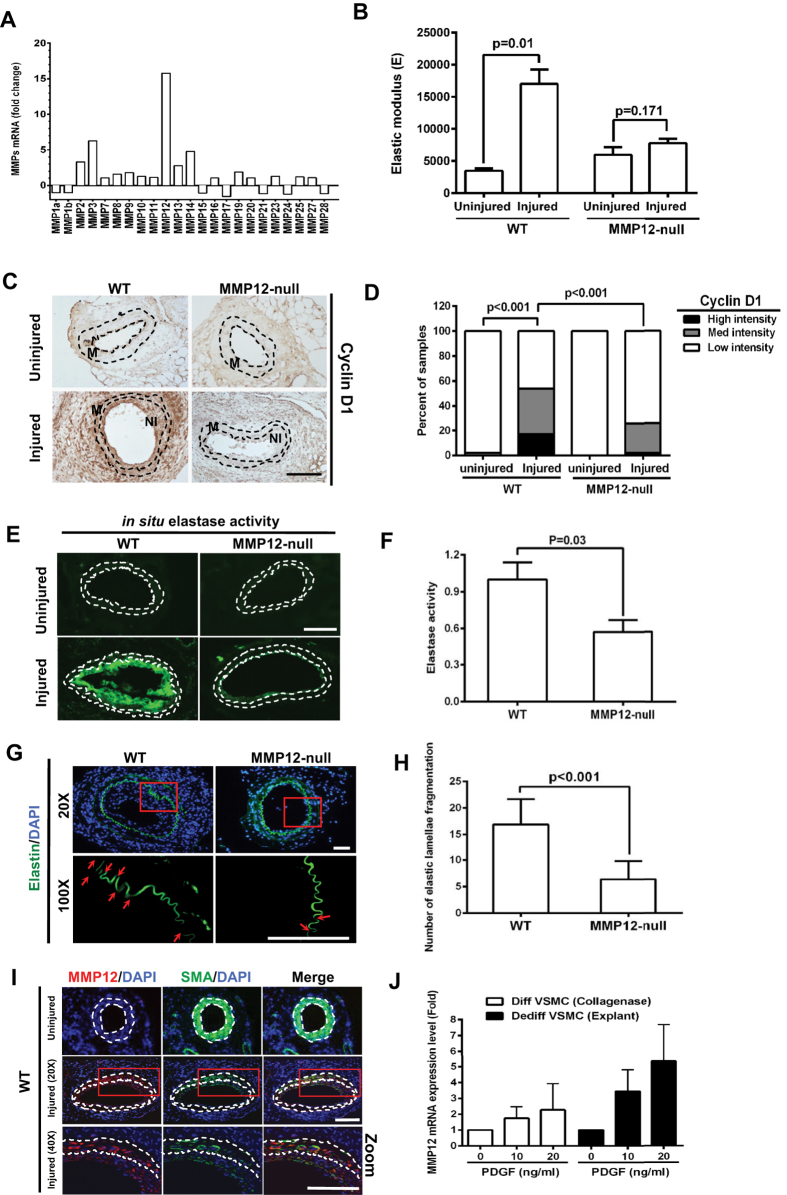
Preferential induction of MMP12 is causal for arterial stiffening after vascular injury. Male wild-type and MMP12-null mice were subjected to femoral artery injury. (**A**) Differential expression of MMP mRNAs after femoral artery injury in 4–5 month old wild-type mice. (**B**) Uninjured (n = 6) and injured (n = 4) femoral arteries from wild-type and MMP12-null mice were isolated, cleaned, and immediately analyzed by AFM. The bar graph shows mean + SE. (**C**) Cyclin D1 staining of uninjured and injured femoral artery sections. Dashed lines show the internal elastic lamina (IEL) and external elastic laminae (EEL). M; media. NI; neointima. Scale bar = 50 μm. (**D**) Blind quantification of results in C from wild-type (n = 10) and MMP12-null (n = 8) mice. Statistical significance was determined by chi-square test. (**E**) *In situ* elastase activity of cross sections of uninjured and injured femoral arteries. Scale bar = 50 μm. (**F**) Quantification of results in panel E from wild-type (n = 7) and MMP12-null (n = 5) mice. The bar graph shows mean + SE. (**G**) Elastin fragmentation in the IEL was detected by autofluorescence imaging (green). Scale bar = 25 μm. Nuclei (blue) were stained with DAPI. (**H**) Quantification of results in panel G from wild-type (n = 10) and MMP12-null (n = 8) mice. The bar graph shows mean + SE. (**I**) Cross sections of uninjured and injured femoral arteries of wild-type mice immunostained for MMP12 (red) and SMCs (anti-SMA, green) (n = 8). Nuclei (blue) were stained with DAPI. Dashed lines show the IEL and EEL as determined by autofluorescence. (**J**) Differentiated and de-differentiated SMCs were serum-starved and incubated with 1 mg/ml heat-inactivated, fatty acid-free BSA in the absence (control) or presence of PDGF for 24 h. MMP12 mRNA levels were determined by RT-qPCR and plotted relative to 18S rRNA. The bar graph shows mean + SD; n = 4. The levels of MMP12 mRNA in the absence of PDGF was set to 1.0. All *p* values are from two-tailed Mann-Whitney tests.

We previously showed that arterial stiffness is increased acutely, ~5–10 fold, during the response to femoral artery injury in C57BL/6 mice^9^, and we performed a genome-wide differential expression analysis of these injury sites^8^,^17^. We examined this data set for the differential expression of ECM and ECM-modifying mRNAs in an effort to identify a dynamic upstream inducer of arterial stiffening amongst the many gene products that have the potential to remodel the ECM (Table S1). We found a striking induction of MMP12 mRNA in injured vs. uninjured arteries, and this induction exceeded the differential gene expression of other MMPs (Fig. 1A), including MMP2 and MMP9, which have been implicated in tissue stiffening^18^. The preferential increase in MMP12 mRNA was confirmed by RT-qPCR (Fig. S1A).

To test the importance of MMP12 in this model of acute arterial stiffening, we compared the response to wire injury in femoral arteries of wild-type and MMP12-null mice. As determined by atomic force microscopy (AFM), basal arterial stiffness was similar in wild-type and MMP12-null mice, but the increase in arterial stiffness seen after injury in wild-type mice was absent in the MMP12-null mice (Fig. 1B). A stiff ECM is required for SMC cell cycling^9^,^10^, so the reduced stiffness of MMP12-null injury sites suggested that the proliferative response to injury might be attenuated in MMP12-null mice. Indeed, we found that cyclin D1 levels (Fig. 1C,D), Ki67 staining (Fig. S2A-B), and luminal stenosis (Fig. S2C-D) were all reduced in the injured arteries of MMP12-null mice. Thus, MMP12 promotes arterial stiffening and the SMC injury response.

MMP12 is a canonical elastase^19^,^20^. Elastin supports arterial recoil and cooperates with fibrillar collagens to regulate arterial stiffness^21^,^22^. Thus, the coincidence between increased MMP12 and arterial stiffness suggested that MMP12 might result in arterial stiffening by degrading elastin. We therefore examined *in situ* elastase activity and endogenous elastin fragmentation in wild-type and MMP12-null arteries after injury. Elastase activity (Fig. 1E,F) and endogenous elastin fragmentation (Fig. 1G,H) were reduced 45–65%, respectively, in injured arteries of MMP12-null mice as compared to injured wild-type controls. We note that MMP3, cathespin S (Ctss), and neutrophil elastase (Elane) can also degrade elastin and that the mRNAs for these enzymes were somewhat induced after vascular injury, though well below the level of induction seen for MMP12 mRNA (Fig. 1A, Table S1, and Fig. S1B). These results, coupled with the relatively low amount of residual elastase activity and elastin fragmentation in the injured arteries of MMP12-null mice (Fig. 1E–H), lead us to conclude that MMP12 is a major elastase in this model of acute arterial stiffening.

Tissue stiffening is canonically viewed as a consequence of an increase in the levels of crosslinked fibrillar collagens^8^,^23^,^24^. However, the differential expression of the mRNAs for collagens and lysyl oxidases was much less dramatic than that of MMP12, as was the differential expression of elastin, fibrillin, and fibulin mRNAs (Table S1). Additionally, neointimal collagen-I abundance (determined by immunofluorescence microscopy) and collagen structure (determined by second harmonic generation, SHG, two-photon microscopy) were similar in the media and neointimas of injured arteries of wild-type and MMP12-null mice (Fig. S2E). These data are consistent with a model in which a change in the abundance of intact elastin rather than fibrillar collagen mediates arterial stiffening after injury. They also indicate that the stiffness-regulatory effect of MMP12 is independent of its ability to activate collagenases such as MMP2^25^.

MMP12 was identified in macrophages^19^,^20^, and macrophages can be found during the early inflammatory stage of the response to vascular injury^26^,^27^. However, the appearance of macrophages at the injury site is transient, and we did not detect macrophages (as determined by anti-CD68 immunostaining) in the 14-day injured arteries that strongly expressed MMP12 (Fig. S3A). Two reports indicate that MMP12 is expressed in vascular SMCs^28^,^29^. We did not detect MMP12 in the SMC-rich medial layer of uninjured arteries, but MMP12 staining was associated with weak SMA staining in both the media and neointima of wild-type femoral arteries 14 days after fine-wire injury (Fig. 1I).

The inverse relationship between MMP12 and SMA staining suggested that MMP12 was being expressed by dedifferentiated SMCs. Indeed, we found that differentiated vascular SMCs in culture strongly expressed SMA but did not express MMP12 mRNA (Fig. 1J) or protein (Fig. S3B) either basally or after mitogenic stimulation with PDGF. In contrast, dedifferentiated SMCs weakly expressed SMA but strongly expressed MMP12 in response to PDGF (Fig. 1J and S3B). Others have shown that the MMP12 promoter is activated by the binding of the transcription factor, AP1^29^. Consistent with this result, a selective AP1 inhibitor blocked PDGF-dependent MMP12 mRNA induction in SMCs (Fig. S4). We conclude that a mitogen-sensitive induction of MMP12 in dedifferentiated vascular SMCs leads to high level MMP12 expression during the response to vascular injury.

### Smooth muscle MMP12 and chronic arterial stiffening with age

**Figure 2:**
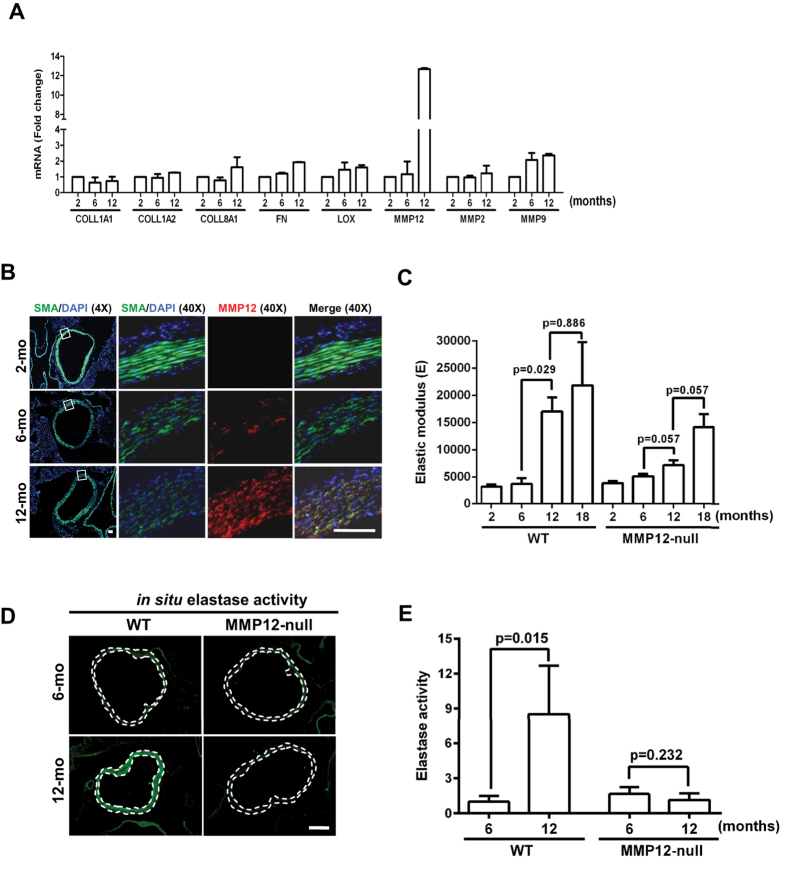
MMP12 mediates age-dependent arterial stiffening. Aortas were isolated from male wild-type and MMP12-null mice of different ages. (**A**) The samples were analyzed by real-time qPCR. Results show mean + SD; n = 2 with each independent experiment representing a pool of 4 aortas. The levels of each mRNA in the 2-month arteries were set to 1.0. (**B**) Cross sections of aortic roots from 2 (n = 7), 6 (n = 8) and 12 (n = 8) month-old male wild-type mice were stained for SMA (green) and MMP12 (red). Scale bar = 50 μm. (**C**) AFM of aortas from 2–18 month-old wild-type (n = 4) and MMP12-null (n = 4) mice. The bar graph shows mean + SE. (**D**) *In situ* elastase activity in cross sections of aortic roots from 6 and 12 month-old wild-type and MMP12-null mice. Scale bar = 50 μm. (**E**) Quantification of results in panel D from wild-type (n = 6) and MMP12-null (n = 8) mice. The bar graph shows mean + SE. All *p* values are from two-tailed Mann-Whitney tests.

Arteries stiffen chronically with age^30^ and remodeling of the arterial ECM by SMCs plays a major role in this process because SMCs are the most abundant arterial cell type. We analyzed the mRNA and protein expression levels of MMP12 in 2, 6, and 12 month-old wild-type mice by RT-qPCR and immunofluorescent staining. The results showed an age-dependent induction of MMP12 occurring at 12 months (Fig. 2A,B). This increased expression of MMP12 was associated with a decreased expression of SMA (Fig. 2B). Macrophages were not detected in these aged arteries (Fig. S5A).

The kinetics of MMP12 mRNA and protein induction (Fig. 2A,B) agreed well with the time course of arterial stiffening (Fig. 2C) in wild-type arteries. Moreover, deletion of MMP12 attenuated age-dependent arterial stiffness (Fig. 2C) and elastase activity (Fig. 2D,E). These results indicate that MMP12 is a major elastase regulating chronic arterial stiffening. As with the response to injury, collagen-I levels (Fig. S5B) and structure (Fig. S5C) were similar in the aged arteries of wild-type and MMP12-null mice. In addition to demonstrating the essential role for MMP12 in a model of chronic arterial stiffening, the minimal arterial stiffening seen in 12-month old MMP12-null arteries indicates that the acutely reduced stiffening we observe in injured arteries of MMP12-null mice (refer to Fig. 1) is not merely a secondary consequence of their reduced response to injury.

### Mechanosensitivity of wild-type and MMP12-null smooth muscle cells

**Figure 3:**
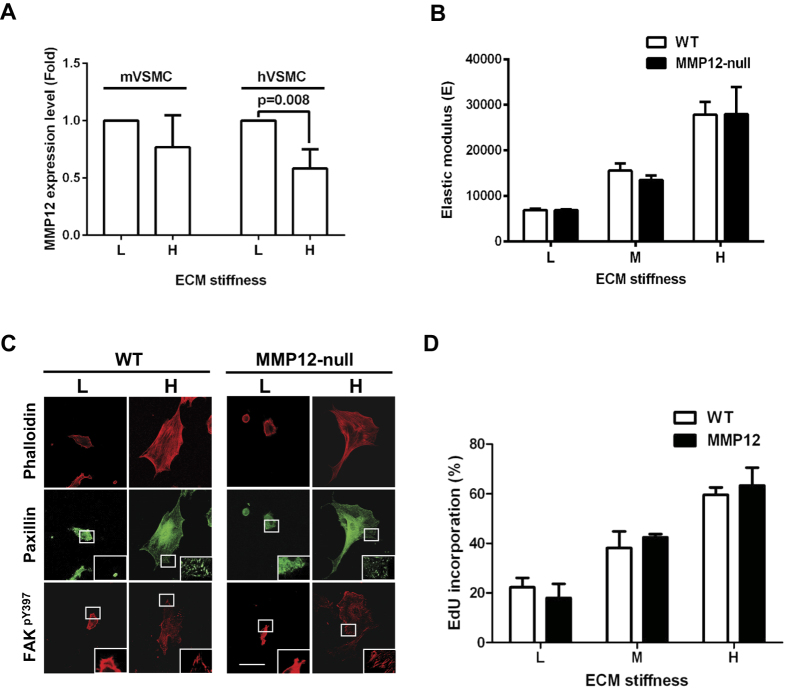
Similar effects of ECM stiffness on wild-type and MMP12-null SMCs in culture. (**A**) Mouse (n = 4) and human (n = 5) vascular SMCs were serum-starved for 48 h and then seeded on low and high stiffness fibronectin-coated acrylamide hydrogels with 10% FBS for 24 h. The bar graph shows mean + SD with the level of MMP12 mRNA in the low stiffness hydrogels set to 1.0. (**B–D**) Wild-type and MMP12-null SMCs were serum-starved and incubated on low (L)-, medium (M), or high- (H)-stiffness hydrogels (2–4, 10–12, and 20–25 kPa, respectively) with 10% FBS for 24 hr. (**B**) Intracellular stiffness was determined by AFM. The bar graph shows mean + SE of 4 independent experiments with 10 cells analyzed per experiment. (**C**) Cells were co-stained with phalloidin (red) and anti-paxillin (green). Replicate coverslips were stained with anti-FAK^PY397^ (red); n = 4 independent experiments with 5 cells analyzed per experiment. (**D**) S-phase entry was determined by EdU incorporation after a 72-hr incubation in 10% FBS. The bar graph shows mean + SD; n = 4. *p* values are from two-tailed Mann-Whitney tests.

Our *in vivo* results above support a model in which MMP12 induction in SMCs leads to ECM remodeling and arterial stiffening, but they do not determine if MMP12 induction is also the consequence of a stiffened ECM. To address this issue, we cultured primary mouse and human aortic SMCs on ECM-coated acrylamide hydrogels set to the stiffness of healthy (2–4 kPa) or injured (20–25 kPa) arteries^9^. MMP12 levels were not increased when wild-type SMCs were cultured on the high- rather than low-stiffness hydrogels (Fig. 3A), indicating that increased MMP12 gene expression is a cause and not a consequence of ECM stiffening.

We also used these deformable substrata to compare the intrinsic mechanoresponisiveness of wild-type and MMP12-null SMCs. The isolated wild-type and MMP12-null cells responded similarly to changes in ECM stiffness as judged by increases in intracellular stiffness (Fig. 3B), the formation actin stress fibers and focal adhesion proteins paxillin and FAK^pY397^ (Fig. 3C), and cell proliferation (Fig. 3D). Additionally, we could show that stiffness-dependent changes in the level of FAK^pY397^ are directly proportional to changes in cellular stiffness as determined by AFM (Fig. S6). Thus, these results show that wild-type and MMP12-null cells similarly sense and respond to changes in ECM stiffness despite the striking differences in the stiffness of the wild-type and MMP12-null arteries. Our interpretation of these results is that the stimulatory effect of MMP12 on arterial stiffness *in vivo* reflects the effect of MMP12 remodeling of the local SMC ECM rather than a change in the mechanoresponsiveness of the SMCs themselves.

### MMP12 is causal for SMC stiffening in mice and predicts SMC stiffness in humans

**Figure 4:**
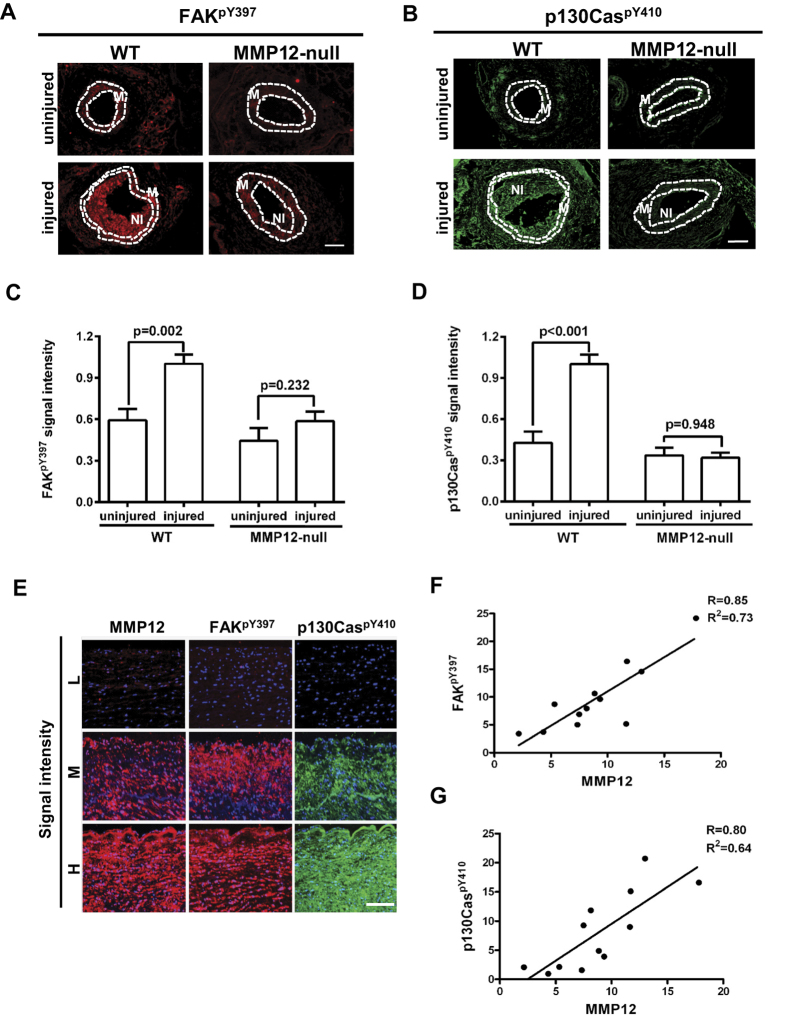
MMP12 expression predicts SMC stiffness. (**A,B**) Cross sections of uninjured and injured femoral arteries of male wild-type [uninjured (n = 8) and injured (n = 10)] and MMP12-null [uninjured (n = 8) and injured (n = 8)] mice were immunostained for FAK^pY397^ and p130Cas^pY410^. Dashed lines show the internal and external elastic laminae as determined by autofluorescence. M; media. NI; neointima. Scale bar = 50 μm. (**C,D**) Quantification of results from panels (**A,B**). The bar graph shows mean + SE. **(E)** Single cross sections of human ascending aortas (n = 12) immunostained for MMP12, FAK^pY397,^ or p130Cas^pY410^. Results from three different patients with low (L), medium (M), and high (H) MMP12 staining are shown. (**F,G**) Linear regression analysis of all patients (n = 12) after immunostaining for MMP12 and either FAK^pY397^ or p130Cas^pY410^.

ECM stiffness is transduced into intracellular stiffness through a signaling pathway involving the phosphorylation of FAK at Y397 and the adaptor protein p130Cas at Y410^10^,^31^,^32^. We therefore compared the abundance of FAK^pY397^ and p130Cas^pY410^ in the injured femoral arteries of wild-type and MMP12-null mice as molecular measures of arterial stiffness to complement the data we obtained by AFM. Consistent with our AFM results, the levels of these molecular stiffness sensors were reduced in paraffin sections of injured (soft) arteries of MMP12-null mice as compared to injured (stiff) wild-type controls (Fig. 4A–D). Thus, MMP12-dependent alterations in ECM can be detected as altered SMC stiffness.

Since the molecular analysis of stiffness by FAK^pY397^ and p130Cas^pY410^ immunostaining is compatible with the analysis of archival tissues, we used FAK^pY397^ and p130Cas^pY410^ abundance to determine if MMP12 expression could predict intracellular stiffness in humans. Indeed, immunostaining of paraffin sections of human ascending aortas showed that MMP12 staining was associated with SMA staining (Fig. S7A). Leukocytes were not detected as assessed by staining for CD45 or CD68 (Fig. S7B). Adjacent sections were then immunostained for FAK^pY397^ or p130Cas^pY410^. MMP12 levels strongly correlated with intracellular stiffness as judged by FAK^pY397^ or p130Cas^pY410^ staining, with correlation coefficients ≥0.8 (Fig. 4E–G). Note that ECM stiffening increases the levels of phospho-FAK and phospho-Cas, but the levels of total FAK and p130Cas protein are not strongly stiffness sensitive^10^. Consistent with these results, we did not see a strong correlation between the expression of MMP12 and either total FAK or total p130Cas in injured mouse femoral arteries (Fig. S8A-D) or human aortas (Fig. S8E-G). Collectively, these results show that the level of MMP12 predicts the degree to which human arterial SMCs have stiffened.

## Discussion

Tissue stiffness is thought to reflect the combined effects of fibrillar collagens and elastin^21^. We previously reported that an increased abundance of crosslinked fibrillar collagen-I contributes to the increased arterial stiffness seen in mice lacking apolipoprotein E^8^. However, arterial stiffening also occurs in the presence of apoE, suggesting that there must be a more global regulator of arterial stiffness. Using both mechanical and molecular measures of arterial stiffness, the experiments described here indicate that this global regulator is MMP12. Although the regulation of MMP activity can be controlled transcriptionally, translationally and post-translationally, changes in the levels of MMP12 protein and enzymatic activity that we measured during both acute and chronic arterial stiffening always agreed with the changes in MMP12 mRNA.

As MMP12 is a canonical elastase, our results suggest that both acute and chronic arterial stiffening can be guided, not only by an increase in fibrillar collagen, but by a decrease in the abundance of intact elastin. Nevertheless, we cannot formally exclude the possibility of MMP12 regulating arterial stiffness through other, elastin-independent, mechanisms^33^,^34^ because the elastin-null mouse is a perinatal lethal^35^ and the use of a conditionally-expressed floxed *ELN* allele is confounded by the restriction of elastin gene expression to embryonic and perinatal life^21^,^36^. Elastin fragmentation may also have an indirect effect on arterial stiffness through the generation of bioactive fragments^37^. It is notable, however, that neonatal *ELN*-null mice have stiff arteries and die of SMC hyperplasia^35^,^38^, arterial phenotypes completely consistent with the reduced stiffness and SMC hyperplasia we see in MMP12-null arteries where elastin degradation is strongly reduced. We did observe a relatively small and delayed increase in arterial stiffness in MMP12-null arteries at 12–18 months. This delay in stiffening may reflect the action of alternative elastases or a more complex remodeling of the aged ECM and is a matter for future study.

A limitation of this study is that our mechanical measurements of arterial stiffness were determined *ex vivo* by AFM. The AFM indented into the intimal cells, but several studies including those shown here demonstrate that cells sense substratum stiffness and adjust their intracellular stiffness accordingly^39^,^40^. Thus, this approach provides a surrogate measure of substratum stiffness. Nevertheless, we have previously used AFM to reveal changes in arterial stiffness that correspond well to changes in the content of fibrillar, structured collagen^8^. AFM also measures small regions of tissues, but we regularly performed the AFM analysis at multiple random areas through the samples under study in an effort to obtain an assessment of overall tissue stiffness. Our results with tissue AFM also agree well with assessment of SMC stiffness using the molecular markers, phospho-FAK and phospho-p130Cas.

Macrophage MMP12 is intimately involved in the inflammatory component of atherosclerosis and chronic obstructive pulmonary disease^25^,^41^,^42^,^43^,^44^, but we did not detect macrophages in the stiffened sites of vascular injury, the aged mouse arteries, or the human archival tissues. In each case, immunostaining for MMP12 was associated with weak SMA staining, suggesting that MMP12 was being induced in dedifferentiated SMCs, a point we confirmed by direct analysis of freshly isolated SMCs. The role of MMP12 expressed by SMCs has not been well understood. Our data indicate that the induction of MMP12 in dedifferentiated SMCs contributes to the epidemiologic relationships between arterial stiffness, age, and CVD.

## Methods

### *In vivo* and *ex vivo* analysis

C57BL/6 (wild-type) and MMP12-null mice on the C57BL/6 background were obtained from Jackson Laboratories (Bar Harbor, ME). Animal work in this study was approved by the Institutional Animal Care and Use Committees of the Wistar Institute and University of Pennsylvania. The methods were carried out in accordance with the approved guidelines. Male mice were aged on a chow diet and, where indicated, subjected to fine-wire vascular injury as described^17^,^45^. Isolated femoral arteries and aortas were analyzed by AFM and stained for elastin, SMA, Ki67, MMP12, FAK^pY397^ and p130Cas^pY410^ as described in Supplemental Methods. Sections of human ascending aortas were obtained through the Gift of Life Donor/Transplant program (courtesy of Ken Margulies) and from the University of Pennsylvania Cardiac Bioregistry (GF), a human repository of surgically resected tissues (IRB protocol # 809349). Patients were 62 ± 6 years of age (mean ± SD; n = 12, 9 of which were female). Immediately upon harvest, aortic tissue was stored in ice-cold DMEM/Ham’s F-12 (Invitrogen), fixed in paraformaldehyde at 4 °C overnight, embedded in paraffin, and cut into 10-μm cross sections.

### SMC isolation and culture

Primary mouse aortic SMCs were isolated from 8–12 weeks old male wild-type or MMP12-null mice. Dedifferentiated SMCs were prepared by explant culture as described previously^46^ and used between passages two to five. Differentiated mouse SMCs were prepared by enzymatic dissociation and short-term (5–7 days) primary cultured as described^8^. All cells were maintained in growth medium (1:1 Dulbecco’s modified Eagle’s medium (DMEM)/Ham’s F-12 supplemented with 2 mM L-glutamine) with 10% FBS. Near confluent monolayers were serum-starved with 1 mg/ml heat-inactivated fatty acid-free BSA for 48 hr before stimulation with FBS or PDGF.

### Bioinformatics

Our analysis of ECM and ECM-remodeling genes differentially expressed after vascular injury is based on data we deposited as GEO dataset, GSE40637. Also see^8^. Log_2_ transformed and quantile normalized expression data were graphed.

### Statistics

Data were analyzed using a two-tailed Mann-Whitney test. Standard Deviation (SD) and Standard Error of the Mean (SE) were used to represent the accuracy of individual values and calculated means, respectively.

## Additional Information

**How to cite this article**: Liu, S.-L. *et al.* Matrix metalloproteinase-12 is an essential mediator of acute and chronic arterial stiffening. *Sci. Rep.* **5**, 17189; doi: 10.1038/srep17189 (2015).

## Supplementary Material

Supplementary Information
