# Supplementary material for: Matrix metalloproteinase-12 is an essential mediator of acute and chronic arterial stiffening
Source: Sci Rep. 2015 Nov 26;5:17189. doi: 10.1038/srep17189 (PMC4660439; doi:10.1038/srep17189)
Supplement: Supplementary Information [file srep17189-s1.pdf]

SUPPLEMENTARY INFORMATION

Matrix metalloproteinase-12 is an essential mediator of acute and chronic arterial stiffening

Shu-Lin Liu[1*], Yong Ho Bae[1], Christopher Yu[1], James Monslow[2], Elizabeth A. Hawthorne[1], Paola Castagnino[1], Emanuela Branchetti[3], Giovanni Ferrari[3], Scott M. Damrauer[3], Ellen Puré[2], and Richard K. Assoian[1*]

Departments of [1]Systems Pharmacology and Translational Therapeutics, [2] Biomedical Sciences, and [3]Surgery, University of Pennsylvania, Philadelphia, PA 19104

*Corresponding author. E-mail: assoian@mail.med.upenn.edu ; shuliu@mail.med.upenn.edu

SUPPLEMENTAL METHODS

Vascular injury. Fine-wire injury was performed on the left femoral arteries of 4-5 month, male wild-type and MMP12-null mice on the C57BL/6 background as described[1,2]. After 14 days, the mice were perfused with PBS, sacrificed, and the segment of the left femoral artery just proximal to the injury was excised, fixed in 3.7% formaldehyde, and embedded in paraffin. Three peak injury sections were stained for elastin using the Accustain Elastic Stain (Sigma-Aldrich). Luminal, medial, and neointimal areas were quantified from 3 near-adjacent peak sections using Image Pro software, and the mean values were used to calculate percent luminal stenosis for each mouse. The right uninjured arteries were used as controls.

Tissue immunostaining. Paraffin-embedded mouse femoral arteries and human ascending aortas were cut into 10-µm sections, deparaffinized and subjected to microwave heat for antigen retrieval in 10 mM citrate buffer (pH 6.0) for 20 min. The sections were then blocked with 3% BSA in PBS for 15 min at room temperature and incubated overnight with a 200-fold dilution of antibodies to MMP12 (ab52897; Abcam), FAK^{pY397} (141-9; Life Technologies), $p130Cas^{pY410}$ (SAB4503824, Sigma-Aldrich), FAK (05-182, Upstate), p130Cas (sc-9052 for mouse tissue or sc-860 for human tissue, Santa Cruz), Ki67 (M7249; DAKO), collagen-I (1310-01, Southern Biotech), or CD68 (ab53444; Abcam). Sections were washed three times in PBS, followed by incubation with Alexa Fluor 594-conjugated goat anti-rabbit IgG (A11012; Invitrogen), Alexa Fluor 488-conjugated chicken anti-rabbit IgG (A21441; Invitrogen), Alexa Fluor 594-conjugated goat anti-mouse IgG (A11005; Invitrogen), rhodamine (TRITC)-AffiniPure F(ab')2 fragment rabbit anti-goat IgG (305-026-003, Jackson ImmunoResearch Laboratories) or Alexa Fluor 594-conjugated goat anti-rat IgG (A11007; Invitrogen) in PBS for 2 hr at room

temperature. Alternatively, the sections were costained with anti-MMP12 and FITC-conjugated anti-α-SMA monoclonal antibody (clone 1A4; Sigma-Aldrich). For the immunostaining analysis of MMP12 in aged mouse arteries, hearts with the ascending aorta were placed in fresh-frozen optimal cutting temperature medium (OCT) as previously described [3]. Frozen cross-sections (10-μm) of the ascending aortas were fixed in 3.7% formaldehyde and washed three times in PBS; staining was done following the aforementioned procedure starting with the BSA blocking step.

For immunohistochemical staining, paraffin sections were blocked with power block buffer (HK083-5K; BioGenex Laboratories; cyclin D1) or 3% BSA in PBS (CD45 and CD68) for 15 min. The sections were then incubated with antibodies to cyclin D1 (Ab-3; Thermo Scientific), CD45 (550539; BD), or CD68 (ab53444; Abcam) overnight at 4°C, washed in PBS, and then incubated for 2 hr with biotinylated goat anti-rat IgG (BH-9400; Vector Laboratories) in PBS. Vectastain ABC (PK-6100; Vector Laboratories) and 3,3′-diaminobenzidine (K3467; Dako) were used to detect the proteins. Images were captured at 4X, 20X and 40X magnification using a Nikon Eclipse 80i microscope equipped with either a QImaging MicroPublisher 5.0 RTV Camera or Hamamatsu C4742-95 digital camera and camera controller. Image processing was with Image J or ImagePro (Media Cybernetics).

Cell immunostaining. Primary differentiated and dedifferentiated VSMCs were fixed in 3.7% formaldehyde, permeabilized with 0.2% Triton X-100 in PBS for 5 min, blocked with 3% BSA in PBS for 15 min at room temperature, and incubated with 200-fold dilutions of anti-MMP12 (ab52897; Abcam) or FITC-conjugated anti-α-SMA (clone 1A4, Sigma-Aldrich). Images were captured at 20X magnification using a Nikon Eclipse 80i microscope equipped with a Hamamatsu C4742-95 digital camera and camera controller and processed with ImagePro

(Media Cybernetics). VSMCs on hydrogels were fixed in 3.7% formaldehyde for 1 hr, permeabilized with 0.5% Triton X-100 for 15 min, and then blocked in 3% BSA for 30 min at room temperature. The samples were stained with Alexa Fluor-594 phalloidin (A12381; Invitrogen), anti-paxillin (sc-5574; Santa Cruz Biotechnology) and anti-FAK^{pY397} (141-9; Life Technologies) in PBS overnight at 4°C, and washed in three times with PBS. Samples stained for paxillin or FAK^{pY397} were then incubated with Alexa Fluor 488–conjugated goat anti-rabbit for 2 hr at room temperature. Species-specific IgG was used as negative control. Images were captured at 40X magnification using a Leica TCS SP5 confocal microscope.

Atomic force microscopy. AFM was adapted from methods previously described[4,5]. Femoral arteries or aortas were isolated from C57BL/6 mice, and visible fat was removed. The cleaned tissues were opened longitudinally, being careful not to disrupt the intima, and placed in a 35-mm tissue culture dish. Excess liquid was removed without touching the tissue. Each end of the tissue was then glued to the culture dish, with the intimal side face-up, using a few microliters of cyanoacrylate adhesive. After ~30 seconds, the glue had set, and the moist sample was fully immersed in 3 ml PBS. Arterial stiffness was determined by indenting into the intima using a DAFM-2X Bioscope AFM (Veeco) in contact mode and silicon nitride AFM probes with a spherical tip (1 μm diameter SiO_2 particle; Novascan). The nominal cantilever spring constant was 0.06 N/m. To calculate the elastic modulus, the first 600 nm of tip deflection was fit with the Hertz model for a sphere. AFM force curves were then analyzed and converted to Young's modulus using custom MATLAB scripts generously provided by Paul Janmey (University of Pennsylvania). AFM results are reported in Pascals.

In an effort to obtain a representative value of arterial stiffness, the AFM analysis was repeated at five randomly chosen regions throughout the artery. Moreover, the stiffness of each of the five sites was determined from 5 replicate force curves taken within a small distance of each other. A mean stiffness was calculated for each of the five sites, and those values were used to calculate a mean stiffness for the tissue as a whole. To eliminate artifacts, force curves showing stiffness >100 kPa (generally less than 10% of total measurements) were not included in the analysis.

The stiffness of isolated VSMCs on FN-coated hydrogels was measured as previously described[5] except that some experiments were performed with a Bruker Catalyst AFM. A standard silicon nitride cantilever (Bruker; nominal spring constant, 0.06 N/m) with a conical tip (40-nm in diameter) was used and the first 600 nm of tip deflection was fit with the Hertz model for a cone.

Elastic lamellae fragmentation. After being deparaffinized and rehydrated, peak cross-sections of injured femoral arteries were stained with DAPI Fluoromount-G (0100-01; Southern Biotech). Autofluorescence images were captured at 100X magnification under oil using a Nikon Eclipse 80i microscope equipped with a Hamamatsu C4742-95 digital camera and camera controller with ImagePro (Media Cybernetics). The number of sites of elastin fragmentation was counted manually for each section, and the mean of three near-adjacent sections was calculated.

In situ zymography. Elastase activity was measured by *in situ* zymography as described[6]. Frozen cryostat sections in OCT (10 µm) were incubated overnight at room temperature in a humidified dark chamber with 40 µg/ml fluorescein (FAM)-conjugated elastin (85113, AnaSpec)

dissolved in Novex zymogram developing buffer (LC2671, Invitrogen). Sections were also incubated with EDTA (20 mM) or buffer only as negative controls. Elastase activity was identified as green fluorescence.

Second harmonic generation (SHG) two-photon microscopy. SHG images were captured with a 20X water dipping lens using a Prairie Technologies Ultima 2-Photon Microscope system (Middleton, WI) as previously described[7]. Images were taken with an excitation wavelength of 910 nm, and captured through emission filters of 457-487 nm (SHG signal) and 525-570 nm (tissue autofluorescence). The SHG and autofluorescence signals were pseudo-colored in green and red, respectively. The remaining green signal after merging both channels is the SHG signal specific for collagen.

Preparation of polyacrylamide hydrogels. Fibronectin-coated polyacrylamide hydrogels were prepared similarly to the methods previously described[5,8]. The acrylamide concentration remained constant at 7.5% and either 0.03, 0.06, 0.15, or 0.3% bis-acrylamide. The hydrogels were prepared on 18-mm (immunostaining and AFM) or 40-mm (immunoblotting) coverslips.

EdU incorporation assay. Serum-starved cells were plated on fibronectin-coated hydrogels with fresh growth medium containing 10% FBS. To assess DNA synthesis, cells were incubated with 10 μM EdU (Invitrogen) for 72 hr in the presence of 10% FBS in DMEM. EdU was visualized using the Click-iT EdU Imaging Kit (Invitrogen). Nuclei were stained with DAPI, and the stained coverslips were mounted on glass slides. Three fields of view (typically 30-60 cells per sample) were counted to determine the percent EdU-positive cells relative to DAPI-stained nuclei.

Immunoblotting. Near-confluent VSMCs were serum-starved by incubation in DMEM/F12 with 1 mg/ml heat-inactivated fatty-acid free BSA for 48 hr. The serum-starved cells were trypsinized, collected by centrifugation, resuspended in serum-free growth medium for 30 min at 37°C, and replated on fibronectin-coated hydrogels with fresh DMEM/F12 medium containing 10% FBS. Cell lysates were prepared, fractionated on 10% reducing SDS polyacrylamide gels and electrophoretically transferred to nitrocellulose filters as described[5]. Filters were blotted with antibodies to FAK ^{pY397} (3283, Cell Signaling) and FAK (610088, BD biosciences). Signals were detected using enhanced chemiluminescence and quantified with ImageJ.

Image analysis. Results from the *in situ* elastase activity and immunostaining of injury sections were quantified using Image J. The region of interest for each immunostained tissue section was delineated using the polygon selection tool; raw integrated density (RawIntDen) and sample areas were obtained. The RawIntDen/area was calculated for each section, and the area-normalized RawIntDen background signal was subtracted based on the mean value obtained from three randomly selected regions without tissue. Unless noted otherwise, the mean net signal from three near-adjacent sections was calculated and graphed.

Reverse transcription quantitative PCR (RT-qPCR). RT-qPCR was performed as described[8] using reverse transcription reactions containing 50 ng or 100 ng of total RNA isolated from cultured VSMCs or aortas, respectively. Ten percent of the cDNA was subjected to qPCR with the following primer-probe sets from Applied Biosystems: mouse MMP-2 (Mm00439496), mouse MMP-3 (Mm00440295;), mouse MMP-9 (Mm00442991), mouse MMP12 (Mm00500554), mouse COL1A1 (Mm00801666), mouse COLL1A2 (Mm00483888), mouse

LOX (Mm00495386), mouse fibronectin (Mm01256744), and mouse CTSS (Mm01255859). The primer-probe set for 18S rRNA has been described[8]. Real-time qPCR results were calculated using the ddCt method with 18S rRNA as the reference.

SUPPLEMENTAL REFERENCES

1. Castagnino P, Kothapalli D, Hawthorne EA, Liu SL, Xu T, Rao S, Yung Y, Assoian RK. miR-221/222 Compensates for Skp2-Mediated p27 Degradation and Is a Primary Target of Cell Cycle Regulation by Prostacyclin and cAMP. *PLoS One*. 2013;8:e56140.

2. Kothapalli D, Zhao L, Hawthorne EA, Cheng Y, Lee E, Pure E, Assoian RK. Hyaluronan and CD44 antagonize mitogen-dependent cyclin D1 expression in mesenchymal cells. *J Cell Biol*. 2007;176:535–544.

3. Baglione J, Smith JD. Quantitative assay for mouse atherosclerosis in the aortic root. *Methods Mol Med*. 2006;129:83–95.

4. Klein EA, Yin L, Kothapalli D, Castagnino P, Byfield FJ, Xu T, Levental I, Hawthorne E, Janmey PA, Assoian RK. Cell-cycle control by physiological matrix elasticity and in vivo tissue stiffening. *Curr Biol*. 2009;19:1511–1518.

5. Bae YH, Mui KL, Hsu BY, Liu S-L, Cretu A, Razinia Z, Xu T, Puré E, Assoian RK. A FAK-Cas-Rac-lamellipodin signaling module transduces extracellular matrix stiffness into mechanosensitive cell cycling. *Sci Signal*. 2014;7:ra57.

6. Johnson JL, Devel L, Czarny B, George SJ, Jackson CL, Rogakos V, Beau F, Yiotakis A, Newby AC, Dive V. A Selective Matrix Metalloproteinase-12 Inhibitor Retards Atherosclerotic Plaque Development in Apolipoprotein E–Knockout Mice. *Arterioscler Thromb Vasc Biol*. 2011;31:528–535.

7. Kothapalli D, Liu SL, Bae YH, Monslow J, Xu T, Hawthorne EA, Byfield FJ, Castagnino P, Rao S, Rader DJ, Pure E, Phillips MC, Lund-Katz S, Janmey PA, Assoian RK. Cardiovascular Protection by ApoE and ApoE-HDL Linked to Suppression of ECM Gene Expression and Arterial Stiffening. *Cell Rep*. 2012;2:1259–1271.

8. Klein EA, Yung Y, Castagnino P, Kothapalli D, Assoian RK. Cell adhesion, cellular tension, and cell cycle control. *Methods Enzym*. 2007;426:155–175.

Supplementary Figure 1

A

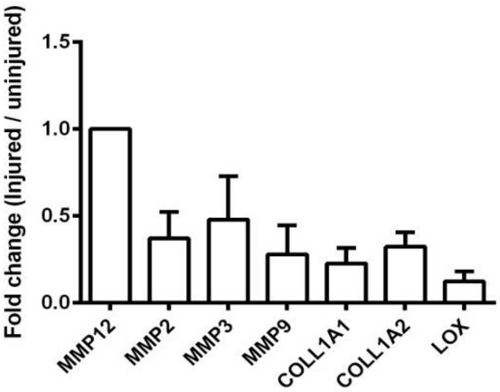

B

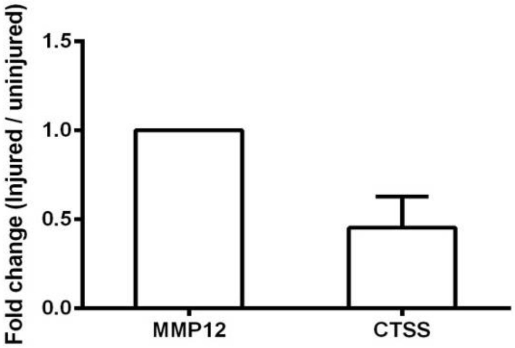

Figure S1. Induction of MMP12 mRNA levels in injured femoral arteries. (A-B) mRNA from microdissected regions of injured femoral arteries of male mice[1] was linearly amplified and then used to quantify several mRNAs by RT-qPCR. The results are plotted relative to linearly amplified RNA from uninjured controls. The bar graph shows mean + SD (n=4) with MMP12 mRNA abundance set to 1.0.

Supplementary Figure 2

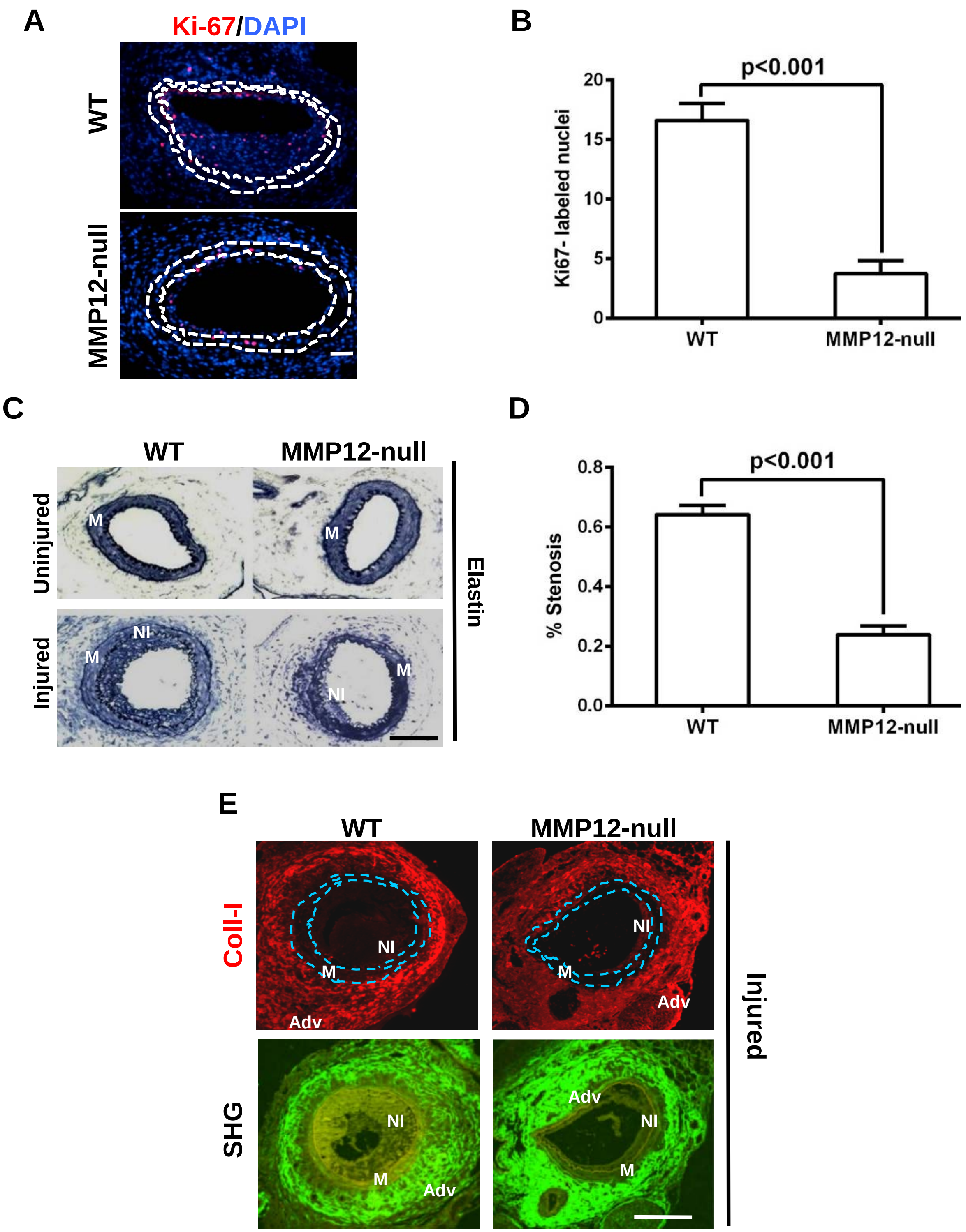

Figure S2. Relative levels of cell proliferation, luminal stenosis and collagen abundance and structure in injured arteries of wild-type and MMP12-null mice. **(A)** Cross sections of injured femoral arteries were stained for Ki67. Scale bar=50 μm. (**B**) Quantification of Ki67 results from wild-type (n=9) and MMP12-null (n=8) mice. The bar graph shows mean + SE. (**C**) Cross sections of uninjured and injured femoral arteries from wild-type and MMP12-null mice were stained for elastin. M; media. NI; neointima. Scale bar=50 μm. (**D**) Quantification of luminal stenosis of injured wild-type (n=10) and MMP12-null (n=8) mice. The bar graph shows mean + SE. *p* values in A-D are from two-tailed Mann-Whitney tests. (**E**) Representative cross section images of injured arteries immunostained for collagen-I (red) in wild-type (n=5) and MMP12-null (n=4) mice. Representative second harmonic generation (SHG) images of structured fibrillar collagen from wild-type (n=8) and MMP12-null (n=3) mice. The SHG and elastin autofluorescence signals are pseudocolored green and red, respectively. Scale bar=50 μm. Adv; adventitia.

Supplementary Figure 3

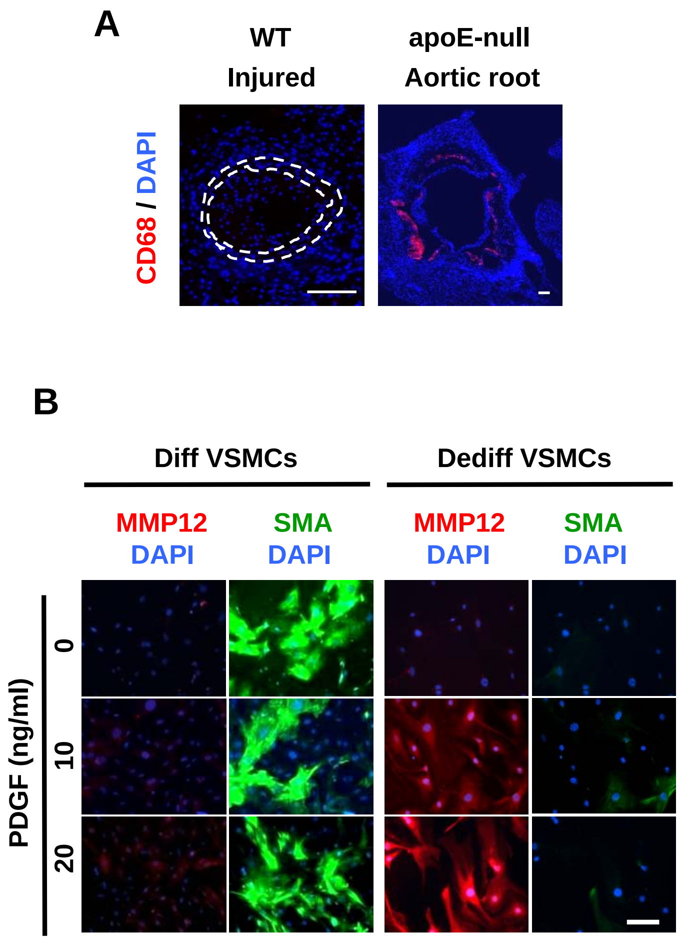

Figure S3. Induction of MMP12 in PDGF-stimulated VSMCs. (A) Cross sections of injured femoral arteries from male wild-type mice were stained for CD68 (n=8). Dashed lines show the IEL and EEL as determined by autofluorescence. An aortic root section of apoE-null mice fed a high-fat diet for 16 weeks was stained for CD68, in parallel, as positive control. Scale bar=50 μm. (**B**) Differentiated or dedifferentiated VSMCs were serum-starved and treated with PDGF as described in the legend for Fig. 1J. Cells on coverslips were co-stained for MMP12 (red), smooth muscle actin (SMA; green), and nuclei (blue). n=4. Scale bars=50 μm.

Supplementary Figure 4

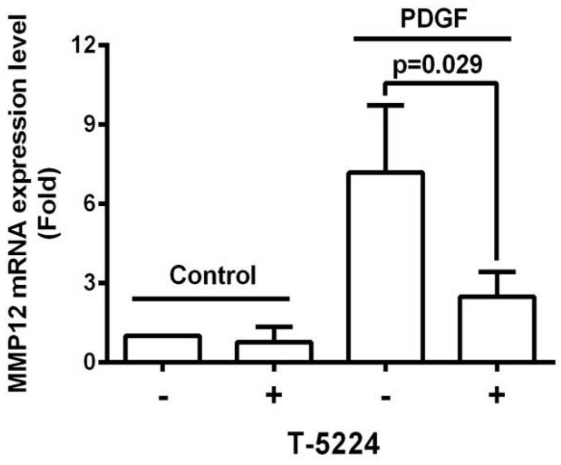

Figure S4. Effect of AP1 inhibition on MMP12 gene expression. Early passage explant cultures of mouse aortic SMCs were grown to near confluence, serum-starved, pretreated T-5224 (AP-1 inhibitor; APExBIO) for 30 min and then stimulated with 20 ng/ml PDGF-BB for 24 hr. MMP12 RNA levels were determined by RT-qPCR. Results show mean + SD, n=4 with the level of MMP12 mRNA in the untreated control cells set to 1.0.

Supplementary Figure 5

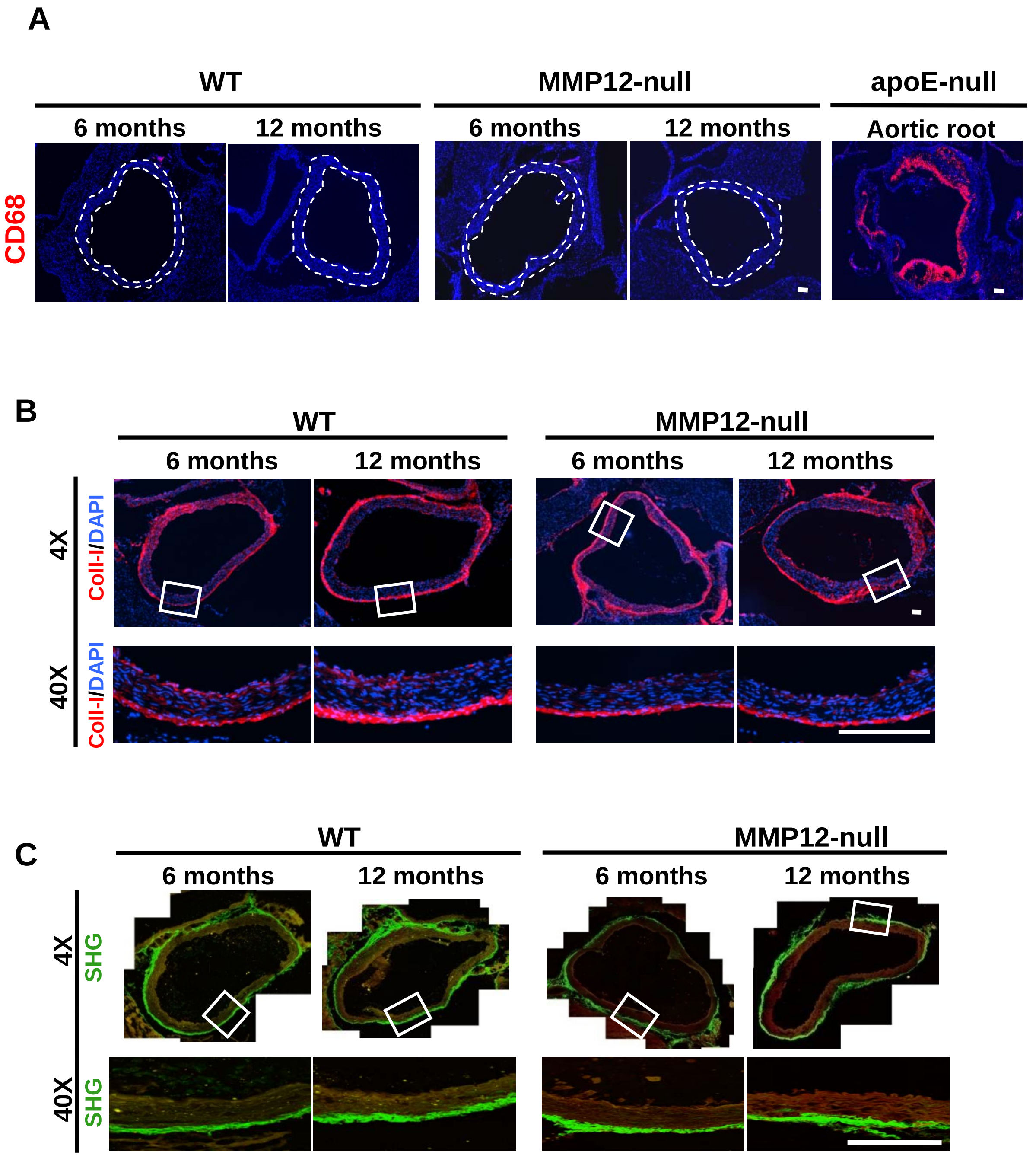

Figure S5. Similar collagen-I abundance and fibrillar collagen structure in the aortas of aging wild-type and MMP12-null mice. (**A**) Cross sections of aortic roots from wild-type and MMP12-null mice at 6 months (n=8 per genotype) or 12 months (n=7 per genotype) of age were immunostained for CD68 (red) and nuclei (DAPI). Dashed lines show the IEL and EEL as determined by autofluorescence. An aortic root section of apoE-null mice fed a high-fat diet for 16 weeks was stained for CD68, in parallel, as positive control. Scale bar=100 μm. **(B)** Cross sections of aortic roots were immunostained for collagen-I (red) and nuclei (DAPI) in wild-type and MMP12-null mice at 6 months (n=8) or 12 months (n=7) of age. Scale bar=100 μm. (**C**) Detection of structured collagen by SHG microscopy of cross-sections from aortic roots of 6- and 12-month wild-type (n=4) and MMP12-null mice (n=4). Scale bar=100 μm. Images in the top panels of C are composites.

Supplementary Figure 6

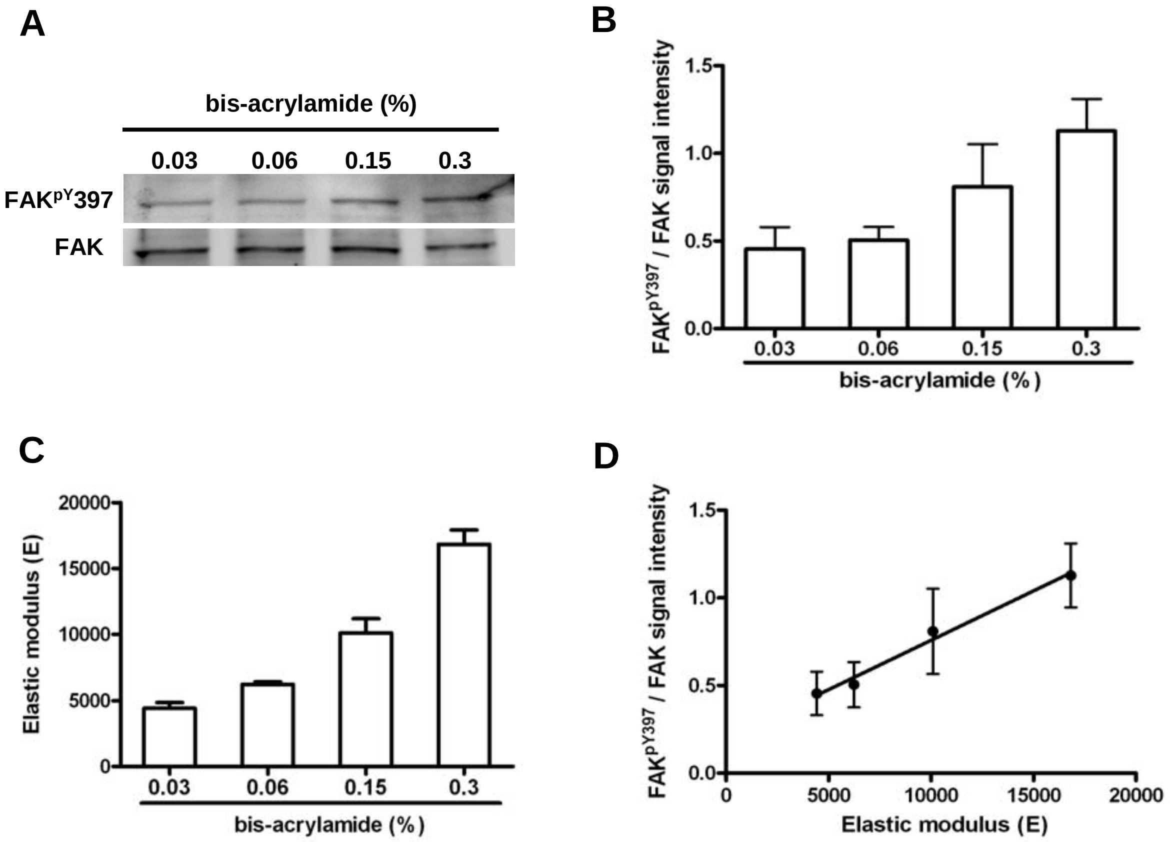

Figure S6. Linear relationship between FAK autophosphorylation and intracellular stiffness. Serum-starved SMCs were suspended in 10% FBS and incubated for 3 hr on fibronectin-coated hydrogels containing 7.5% acrylamide and either 0.03, 0.06, 0.15 or 0.3 % bis-acrylamide. (**A**) Representative immunoblots of total cell lysates probed for phosphorylated and total FAK. **(B)** Signal intensities of FAK^{pY397} and total FAK immunoblots from three independent experiments were quantified with ImageJ and plotted as a ratio. Error bars show SD **(C)** Intracellular stiffness of duplicate cells on hydrogels was determined by AFM. The bar graph shows mean + SE of 3 independent experiments with 6-8 cells analyzed per experiment. **(D**) Linear regression analysis of the results in B and C. Error bars show mean ± SD; n=3. R=0.88, R^2=0.77, and p=0.0002.

Supplementary Figure 7

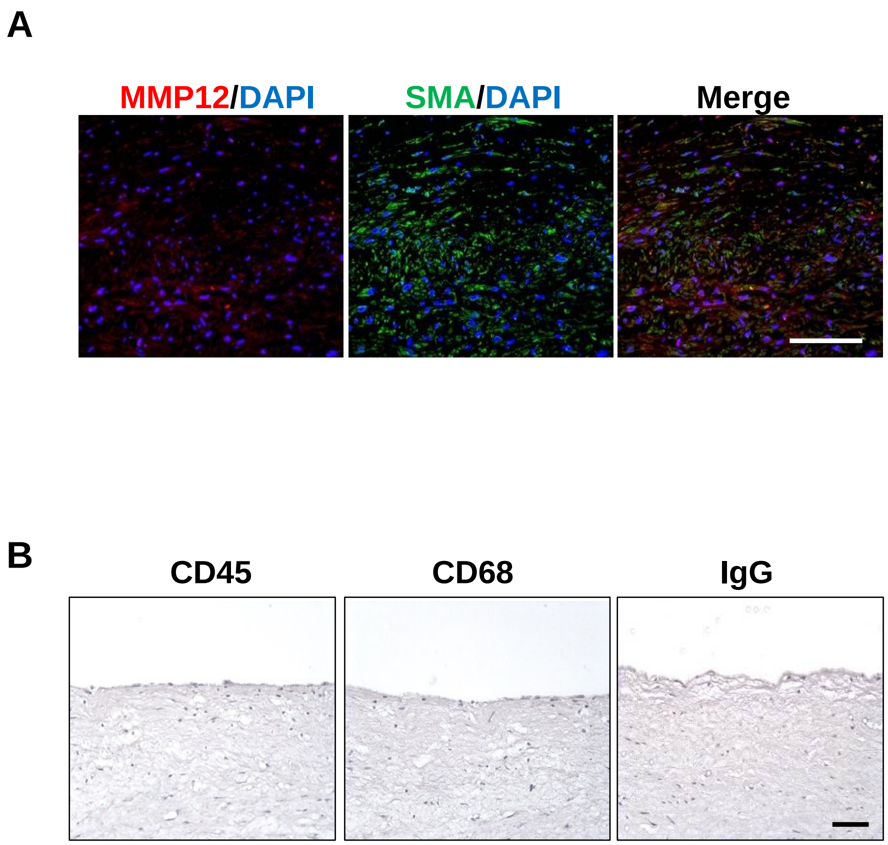

Figure S7. Absence of detectable leukocytes in samples of human ascending aortas. Adjacent cross sections of human ascending aortas were immunostained for either **(A)** MMP12 or SMA (n=12) or **(B)** CD45 and CD68 (n=4). Scale bar=50 μm. IgG was used as the negative control.

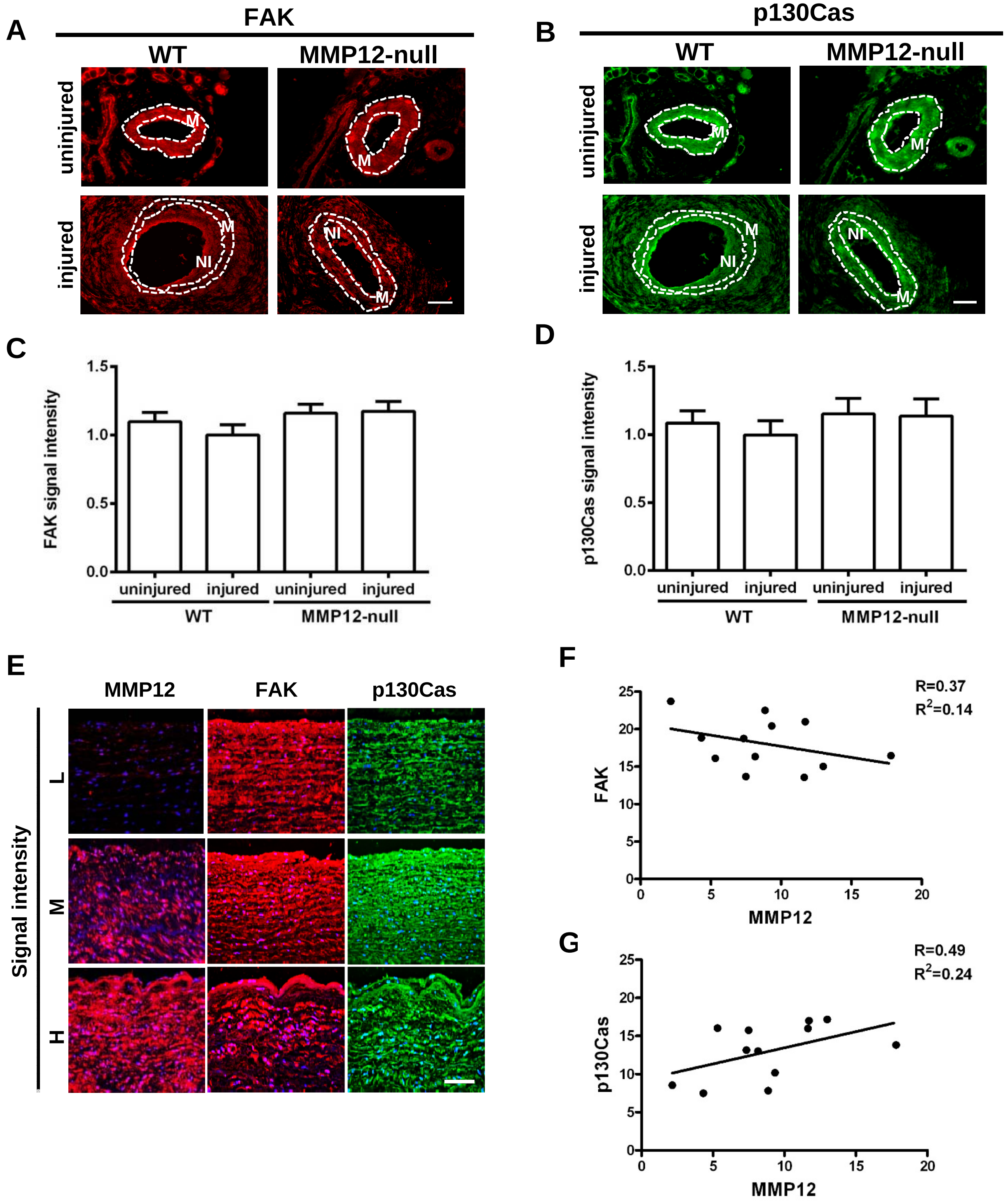

Figure S8. Expression levels of total FAK and p130Cas do not correlate with MMP12 levels. (A-B) Femoral artery cross-sections from male wild-type [uninjured (n=8) and injured (n=10)] and MMP12-null [uninjured (n=8) and injured (n=8)] mice were immunostained for total FAK or total p130Cas. Dashed lines show the IEL and EEL as determined by autofluorescence. M; media. NI; neointima. (**C-D**) Quantification of FAK and p130Cas results in A and B, respectively. The bar graph shows mean + SE. (**E**) Sections of human aortas were immunostained for FAK and p130Cas. DAPI-stained nuclei are in blue. The images were taken from adjacent sections of same aortas used in Fig. 4E, and the MMP12 images from Fig. 4E are reproduced here for reference. (**F-G**) Linear regression analysis for MMP12 and either total FAK or total p130Cas in human aortic sections. Scale bar=50 μm.

Supplementary Table I. Differential Expression of collagens, elastin, MMPs and related genes after vascular injury. Log_2 transformed and quantile normalized expression data are shown. See GSE40637 for primary data.

Gene	Fold change
**Mmp1a**	-1.03504
**Mmp1b**	-1.02803
**Mmp2**	3.29583
**Mmp3**	6.27574
**Mmp7**	1.065
**Mmp8**	1.58262
**Mmp9**	1.80341
**Mmp10**	1.27788
**Mmp11**	1.13132
**Mmp12**	15.7816
**Mmp13**	2.78565
**Mmp14**	4.77538
**Mmp15**	-1.06956
**Mmp16**	1.04953
**Mmp17**	-1.57541
**Mmp19**	1.89812
**Mmp20**	1.07012
**Mmp21**	-1.1728
**Mmp23**	1.30709
**Mmp24**	-1.23465
**Mmp24**	-1.0389
**Mmp25**	1.19404
**Mmp27**	1.09716
**Mmp28**	-1.16552
**Col10a1**	2.31788
**Col11a1**	1.86208
**Col11a2**	-1.04597
**Col12a1**	1.6999
**Col13a1**	-1.29718
**Col14a1**	2.91964
**Col15a1**	1.70408
**Col16a1**	1.04329
**Col17a1**	-1.06238
**Col18a1**	-1.14839
**Col19a1**	-4.23036
**Col1a1**	2.73042
**Col1a2**	2.39269
**Col20a1**	-1.14277
**Col23a1**	-1.03183
**Col24a1**	1.03764

**Col25a1**	-1.10875
**Col27a1**	1.05783
**Col28a1**	1.08813
**Col2a1**	-1.03862
**Col3a1**	2.524
**Col4a1**	1.00789
**Col4a2**	1.06581
**Col4a3**	-1.33334
**Col4a4**	-1.29075
**Col4a5**	-1.56769
**Col4a6**	-1.54134
**Col5a1**	1.36301
**Col5a1**	1.05462
**Col5a2**	2.36337
**Col5a2**	1.14399
**Col5a3**	1.18195
**Col6a1**	1.79194
**Col6a2**	1.66903
**Col6a3**	1.4351
**Col7a1**	-1.04469
**Col8a1**	1.17542
**Col8a2**	1.00598
**Col9a1**	-1.08776
**Col9a2**	-1.05201
**Col9a3**	-1.11432
**Lox**	1.71927
**Loxl1**	1.53784
**Loxl2**	1.6718
**Loxl3**	1.73746
**Loxl4**	1.12685
**Eln**	-1.58403
**Fbn1**	2.73229
**Fbn2**	1.48619
**Fbln1**	-1.05018
**Fbln2**	1.89031
**Fbln5**	-1.18758
**Fbln7**	-1.13867
**Ctss**	7.76467
**Elane**	1.00462
